# Foods and food components in the Mediterranean diet: supporting overall effects

**DOI:** 10.1186/1741-7015-12-100

**Published:** 2014-06-16

**Authors:** Linda C Tapsell

**Affiliations:** 1 Illawarra Health and Medical Research Institute, School of Medicine, University of Wollongong, Wollongong, NSW 2522, Australia

**Keywords:** Mediterranean diet, Cardiovascular disease, Mortality, PREDIMED study

## Abstract

The recent publication of the PREDIMED trial provided definitive evidence that a Mediterranean diet provides protection against cardiovascular disease. Two articles published in *BMC Medicine* provide further understanding of why this may be the case, by considering contributory effects of olive oil, a core food in the diet, and polyphenols, a class of identifiable protective compounds. Using a number of statistical models, analyses were conducted to show around a 35% cardiovascular disease risk reduction in the highest consumers of olive oil and a similar degree of risk reduction for all-cause mortality comparing highest to lowest quintiles of polyphenol intake. The effects were an advance on cohort studies not related to trials. This suggests that it may be necessary to have better control of the background diet to enable exposure of the value of individual foods and nutrients in a dietary pattern, bearing in mind that, by nature, it is difficult to separate out effects of foods, nutrients and whole diets.

Please see related articles: http://www.biomedcentral.com/1741-7015/12/77 and http://www.biomedcentral.com/1741-7015/12/78.

## 

Cardiovascular disease remains a leading cause of death throughout the world. Lifestyle factors including diet, physical activity and smoking habits play a major role in the development and amelioration of disease risk [1,2]. Fifty years ago the links between dietary saturated fat, blood cholesterol and coronary heart disease were introduced, but Keys’ observations valuing the Mediterranean diet in that context may have been overlooked [3]. Later, guidance on the Mediterranean diet highlighted vegetables, legumes, fruits and nuts, cereals, olive oils, fish, with moderate intakes of cheese and yoghurt, low intakes of meat and poultry and regular small quantities of wine with meals [4]. More recently, studies have appeared in the literature providing direct evidence of protective effects.

One of the first substantial observational studies involving Greek adults showed that greater adherence to the Mediterranean diet was inversely associated with death from coronary heart disease (Table 1) [5]. A further analysis found the relative dietary contributions to benefits were moderate alcohol consumption (23.5%), little meat (16.6%), high consumption of vegetables (16.2%), fruits and nuts (11.2%), and legumes (9.7%), and a high dietary monounsaturated fatty acid:saturated fatty acid (MUFA:SFA) ratio (10.6%), [6]. The use of the MUFA:SFA ratio may have been problematic, because it referenced nutrients (fatty acids) rather than foods (such as olive oil) [7]. Olive oil is a characteristic food of the Mediterranean diet, yet by only referring to fatty acid ratios its value could be lost in translation. Later studies remedied the problem.

**Table 1:** **Associations between Mediterranean diet**^**1 **^**or olive oil intake**^**2 **^**and coronary heart disease in European populations**

**Study [reference]**	**Population (age)**	**Age (yr)**	**Number**	**Follow-up (yr)**	**CVD risk (HR; 95% CI)**	***P *** **value**
EPIC Greece [5]	Greek adults	20 to 86	22,043	3.67	0.67 (0.47 to 0.94)	<0.001^1^
EPICOR [8]	Italian women	35 to 74	29,689	7.85	0.56 (0.31 to 0.99)	0.04^2^
PREDIMED trial [9]	Spanish older adults (55 to 80 yr)	55 to 80	7,447	4.8	0.65 (0.47 to 0.89)	0.01^2^
EPIC Spain [10]	Spanish adults (29 to 69 yr)	29 to 69	40,142	10.4	0.78 (0.59 to 1.03)	0.050^2^

^1^Two-point increment in Mediterranean diet score.

^2^Trend for upper versus lower quartile of olive oil intake.

In the EPICOR study of Italian women, olive oil consumption was associated with a reduced risk of coronary heart disease (Table 1) [8]. This was similar to the value for a prospective cohort analysis of the PREDIMED trial reported by Guasch-Ferre *et al.* in *BMC Medicine*[9],which also found a further 4% risk reduction if the olive oil was extra virgin. The PREDIMED study population was a smaller intervention group of older Spanish adults, yet the findings were similar to those obtained from the Spanish EPIC cohort (Table 1). EPIC Spain also showed a greater reduction in risk with virgin olive oil [10], but this difference was not detected in a later period of follow-up (Table 1) [11]. The lack of an association may reflect many things, including a loss of power and greater variation in total dietary intakes.

Plant foods, the basis of the Mediterranean diet, and olive oil in particular, are rich in polyphenols. Processing to produce ordinary olive oil can reduce this attribute, and the bioavailability of these compounds is variable [12]. The polyphenol content of olive oil is important, not only for the delivery of compounds with strong anti-oxidant capacity, but also because it exists in conjunction with fatty acids that are potentially oxidised [12]. Within nutrition science, the critical concept of food synergy recognises that nutrients exist in a purposeful biological sense within foods, delivering them in combinations that reflect biological functionality [3]. Thus, while it is difficult to separate out the effects of foods within a total diet, it is also difficult to study effects of nutrients and bioactive substances in isolation of foods.

Having said that, in the re-analysis of the PREDIMED trial data reported in *BMC Medicine*, Tresserra-Rimbau *et al.*[13] found a 37% relative risk reduction for all-cause mortality between the highest and lowest quintiles of polyphenol intake (HR 0.48; 95% CI 0.25 to 0.91; *P* trend 0.04), adding to another analysis showing a 46% risk reduction of cardiovascular disease (CVD) comparing Q5 and Q1 of polyphenol intake (HR 0.54; 95% CI 0.33 to 0.91; *P* trend 0.04) [14]. The most abundantly consumed polyphenol and phenolic group were 5-caffeoylquinic acid and hydroxycinnamic acids, found in coffee, and the main food sources at baseline comprised coffee, oranges, apples, olives/olive oil and red wine, indicating their significance. However, when compared to other countries, the consumption of olives and olive oil was the main differentiating factor for the Spanish cohort in the consumption of polyphenols [15]. There are other nuances to consider. Total polyphenol intake had a stronger inverse association with mortality risk in women who did not drink alcohol [15], and those beginning with the worst dietary habits more frequently made changes toward better compliance to the diet [16], a phenomenon that has been shown elsewhere [17]. The re-analysis of the PREDIMED trial data reported in *BMC Medicine*[13] found no apparent modification of the polyphenol effect between diet groups, but then the trial was not designed to test the effects of polyphenol intakes. Trials such as PREDIMED produce a wealth of data, but re-analyses demand an appreciation of primary outcomes and sophisticated statistical analyses, all of which have been demonstrated in the papers reported here.

As Ros [7] correctly points out, despite adjustments for confounders such as education, adiposity and diet quality, observational (cohort, case control and cross sectional) studies and analyses cannot infer causality given the possibility of residual confounding. Large-scale trials are required. The PREDIMED study was a major trial comparing the effects of a Mediterranean diet supplemented with olive oil or nuts to a control low-fat diet. The primary results were outstanding, showing a 30% reduction in cardiovascular events in the treatment arms, proving the effect of the diet [18]. There is some discussion as to whether the PREDIMED study tested the effects of the Mediterranean diet or the supplemental foods (olive oil and nuts) [19]. The recent analyses of the PREDIMED study [9,13] address this concern in part by confirming associations with key foods and food components, showing that it is all these influences occurring together. Different forms of analysis on a single trial are informative, as they help to clarify critical elements. This is particularly the case in nutrition science where it is difficult to separate out effects of foods, nutrients and whole dietary patterns [20]. The main take-home message is that the diet is effective and the foods are readily identifiable, but further analyses can be of value.

Not only were the participants in the PREDIMED study at high CVD risk, but they also received healthy dietary advice (both intervention and control). The strength of the food-based analysis reported by Guasch-Ferre [9] is that it distinguishes effects that could be attributed to olive oil, a food that is clearly at the centre of the Mediterranean diet. It may not have been possible to do so if the background diet were much more variable, as may be the case in non-trial cohorts. The lack of an association in the control and the presence of an association in both MedDiet groups (extra virgin olive oil and nut supplemented) seem to confirm this position. The same can be said of the findings reported by Tresserra-Rimbau *et al.*[13]. It may also explain the inability to observe significant associations in observational studies, although developments in statistical approaches are proving very helpful. It may be necessary to have better control of the background diet to be able to appreciate the value of individual foods and nutrients in a dietary pattern. The re-analyses of data from the PREDIMED trial cohort throw light on many issues. Most importantly, they help us better understand the primary outcomes of the trial, and like all good research, they raise further questions for separate investigation.

## Abbreviations

CVD: cardiovascular disease; CI: confidence interval; HR: hazard ratio; MUFA:SFA: monounsaturated fatty acid:saturated fatty acid.

## Competing interests

The author declares that she has no competing interests.

## Author information

LT is a Professor of Nutrition, with undergraduate qualifications in biochemistry and pharmacology, postgraduate qualifications in dietetics and medical education, and a Ph.D. in nutrition and public health. She has conducted and published many dietary intervention trials, researching the effects of dietary fatty acids, various individual foods and healthy dietary patterns. She is a Fellow of the Dietitians Association of Australia and has served on numerous government and non-government organisations relating to nutrition science and policy development.

## References

[B1] Stampfer MJ Hu FB Manson JE Rimm EB Willet WC Primary prevention of coronary heart disease inwomen through diet and lifestyle N Engl J Med 2000 343 16 22 10882764 10.1056/NEJM200007063430103

[B2] Mente A de Koning L Shannon HS Anand SS A systematic review of the evidence supporting a causal link between dietary factors and coronary heart disease Arch Intern Med 2009 169 659 669 19364995 10.1001/archinternmed.2009.38

[B3] Jacobs DR Jr Gross MD Tapsell LC Food synergy: an operational concept for understanding nutrition Am J Clin Nutr 2009 89 1543S 1548S 19279083 10.3945/ajcn.2009.26736B PMC2731586

[B4] Willet WC Sacks F Trichopoulou A Drescher G Ferro-Luzzi A Helsing E Tricholoulos D Mediterranean diet pyramid: a cultural model for eating Am J Clin Nutr 1995 61 1402S 1406S 7754995 10.1093/ajcn/61.6.1402S

[B5] Trichopoulou A Costacou T Bamia C Tricholoulos D Adherence to the Mediterranean diet and survival in a Greek population N Engl J Med 2003 348 2599 2608 12826634 10.1056/NEJMoa025039

[B6] Trichopoulou A Bamia C Tricholoulos D Anatomy of health effects of Mediterranean diet: Greek EPIC prospective cohort study BMJ 2009 338 b2337 19549997 10.1136/bmj.b2337 PMC3272659

[B7] Ros E Olive oil and CVD: accruing evidence of a protective effect BJN 2012 108 1931 1933 10.1017/S0007114512003844 22950835

[B8] Bendinelli B Masala G Saieva C Salvini S Calonica C Sacerdote C Agnoli C Grioni S Frasca G Mettiello A Chiodini P Tumino R Vineis P Palli D Panico S Fruit, vegetables and olive oil and risk of coronary heart disease in Italian women: the EPICOR study Am J Clin Nutr 2011 93 275 283 21177799 10.3945/ajcn.110.000521

[B9] Guasch-Ferre M Hu FB Martinez-Gonzalez MA Fito Montserrat F Bullo M Estruch R Ros E Corella D Recondo J Gomez-Gracia E Fiol M Lapetra J Serra-Majem L Munoz MA Pinto X Lamuela-Raventos RM Basora J Buil-Cosiales P Sorli JV Ruiz-Gutierrez V Alfredo Martinez J Salas-Salvado J Olive oil intake and risk of cardiovascular disease and mortality in the PREDIMED study BMC Med 2014 12:78 10.1186/1741-7015-12-78 PMC4030221 24886626

[B10] Buckland G Travier N Barricarte A Ardanez A Moreno-Iribas C Sanchez MJ Molina-Montes E Chirlaque MD Huerta JM Navarro C Redondo ML Amiano P Dorronsoro M Larranaga N Gonzalez CA Olive oil intake and coronary heart disease in the European Prospective Investigation into Cancer and Nutrition (EPIC) Spanish cohort Br J Nutr 2012 108 2075 2082 23006416 10.1017/S000711451200298X

[B11] Buckland G Mayen AL Agudo A Travier N Navarro C Huerta JM Chirlaque MD Barricarte A Ardanez A Moreno-Iribas C Marin P Quiros JR Redondo ML Amiano P Dorronsoro M Arriola L Molina E Sanchez MJ Gonzalez CA Olive oil intake and mortality within the Spanish population (EPIC-Spain) Am J Clin Nutr 2012 96 142 149 22648725 10.3945/ajcn.111.024216

[B12] Bullo M Lamuela-Raventos R Salas-Salvado J Mediterranean diet and oxidation: Nuts and olive oil as important sources of fat and antioxidants Curr Topics in Medicinal Chem 2011 11 1797 1810 10.2174/156802611796235062 21506929

[B13] Tresserra-Rimbau A Rimm E Medina-Remon A Martinez-Gonzalez MA Carmen Lopez-Sabater M Covas MI Corella D Salas-Salvado J Gomez-Gracia E Lapetra G Arós F Fiol M Ros E Serra-Majem L Pinto X Munoz MA Gea A Ruiz-Gutierrez V Estruch R Lamuela-Raventos RM Polyphenol intake and mortality risk: a re-analysis of the PREDIMED trial BMC Med 2014 12:77 10.1186/1741-7015-12-77 PMC4102266 24886552

[B14] Tresserra-Rimbau A Rimm E Medina-Remon A Martinez-Gonzalez MA de la Torre R Corella D Salas-Salvado J Gomez-Gracia E Lapetra G Arós F Fiol M Ros E Serra-Majem L Pinto X Saez GT Basora J Sorli JV Martinez JA Vinyoles E Ruiz-Gutierrez V Estruch R Lamuela-Raventos RM Inverse association between habitual polyphenol intake and incidence of cardiovascular events in the PREDIMED study Nutr Metab Cardiovasc Dis 2014 http//dx.doi.org/10.1016/j.numecd.2013.12.014 10.1016/j.numecd.2013.12.014 24552647

[B15] Tresserra-Rimbau A Medina-Remon A Perez-Jimenez J Martinez-Gonzalez MA Covas MI Corella D Salas-Salvado J Gomez-Gracia E Lapetra G Arós F Fiol M Ros E Serra-Majem L Pinto X Munoz MA Saez GT Ruiz-Gutierrez V Warnberg J Estruch R Lamuela-Raventos RM Dietary intake and major food sources of polyphenols in a Spanish population at high cardiovascular risk: The PREDIMED study Nutr Metab Cardiovasc Dis 2013 http//dx.doi.org/10.1016/j.numecd.2012.10.008 10.1016/j.numecd.2012.10.008 23332727

[B16] Zazpe I Sanchez-Tainta A Estruch R Lamuela-Raventos R Schröder H Salas-Salvado J Corella D Fiol M Gomez-Gracia E Aros F Ros E Ruiz-Gutierrez V Iglesias P Conde Herrera M Martinez-Gonzalez MA A large randomized individual and group intervention conducted by registered dietitians increased adherence to Mediterranean-type diets: the PREDIMED Study J Am Dietetic Assoc 2008 108 1134 1144 10.1016/j.jada.2008.04.011 18589019

[B17] Grafenauer SJ Tapsell LC Beck EJ Batterham MJ Baseline dietary patterns are a significant consideration in correcting dietary exposure for weight loss Eur J Clin Nutr 2013 doi:10.1038/ejcn.2013.26 10.1038/ejcn.2013.26 23403877

[B18] Estruch R Ros E Salas-Salvadó J Covas MI Corella D Arós F Gomez-Gracia E Ruiz-Gutierrez V Fiol M Lapetra G Lamuela R Serra-Majem L Pinto X Barosa J Munoz MA Sorli JV Martinez JA Martínez-González MA Primary prevention of cardiovascular disease with a Mediterranean diet N Engl J Med 2013 368 1279 1290 29897867 10.1056/NEJMc1806491

[B19] Appel LJ Van Horn L Did the PREDIMED trial test a Mediterranean diet? N Engl J Med 2013 368 1353 1354 23550674 10.1056/NEJMe1301582

[B20] Jacobs DR Tapsell LC Temple NJ Food synergy: the key to balancing the nutrition research effort Public Health Rev 2011 33 509 531

